# Effectiveness of Brief Psychodynamic Therapy With Children and Adolescents: An Outcome Study

**DOI:** 10.3389/fped.2019.00501

**Published:** 2019-12-20

**Authors:** Michela Gatta, Marina Miscioscia, Lorenza Svanellini, Andrea Spoto, Manuela Difronzo, Maxim de Sauma, Emilia Ferruzza

**Affiliations:** ^1^Department of Women's and Children's Health, University of Padua, Padua, Italy; ^2^Department of Developmental Psychology and Socialization, University of Padua, Padua, Italy; ^3^Department of General Psychology, University of Padua, Padua, Italy; ^4^Brent Centre for Young People, London, United Kingdom

**Keywords:** psychodynamic psychotherapy, brief psychotherapy, developmental psychopathology, outcome study, parental support

## Abstract

Studies on the effectiveness of child and adolescent psychotherapy treatments provided by the Italian National Health Service lag behind, while the scientific community has rather focused on the value of cognitive-behavioral psychotherapeutic approaches. This paper evaluates the effectiveness of a one year psychodynamically-oriented intervention with children and adolescents—aged between 6 and 18 years (*M* = 12.08, SD = 3.7)—and their parents, carried out in a Child and Adolescent Neuropsychiatric Service (SCIAF), part of the Italian National Health System. Following a psychodiagnostic assessment, two types of therapeutic intervention were offered: children and adolescents allocated to Group 1 (*N* = 26) were offered individual psychodynamic psychotherapy alone, whilst youths in Group 2 (*N* = 31) were offered individual psychotherapy, accompanied by parental support. This study examines the effects of this time-limited (12 month) psychodynamically-oriented psychotherapy in terms of improvements in patients' symptoms (measured on the Achenbach's questionnaires: *Child Behavior Checklist* and *Youth Self-Report 11-18*). This study also examines the effects of treatment on parents' perception of their family empowerment. This domain is measured on the *Family Empowerment Scale* (FES). Our findings seem to be partly in line with published studies according to which poor parenting (i.e., characterized by lack of warmth, a rigid and/or negative parenting style, poor monitoring of the children, etc.) would be positively associated with Externalizing problems in childhood. Our preliminary findings suggest that brief psychodynamic therapy seemed to show positive outcomes in both “Internalizing” and “Externalizing” difficulties, accounting for age-related differences, ICD-10 (1) diagnoses, and the types of treatment offered. However, no statistically significant changes were detected in the parents' perceptions of empowerment at 12 months.

## Theoretical Background

Psychodynamic psychotherapy focuses specifically on the interactions between the mental processes generated by the person's subjective experiences and the behavior at the onset of such problems (2).

One of its aims is to strengthen the patient's capacity to understand the reasons for their subjective experiences and their underlying meanings, their relationships, and their own and others' behavior (2). The therapist tries to improve the patient's awareness of such unconscious mechanisms and influential factors, and to promote their capacity to tackle overwhelming anxieties and pressures within these relationships (2).

Mental health services are increasingly being asked to provide short-term or time-limited psychodynamic psychotherapies (3). Several models, such as mentalization-based therapy [MBT (4)], dynamic interpersonal therapy [DIT (5)], short-term psychodynamic psychotherapy (6, 7) are now being used in various services.

Short-term psychodynamic psychotherapy, although quite unstructured in its approach, follows some principles (8) to try and draw out a basic understanding of the ongoing determinants of a patient's reported difficulties, crisis or breakdown; overall, it does not seem to primarily focus on the client's past; it rather prioritizes a better understanding of the client's present and current difficulties. Exploration of early years and early relationships is not an aim of this work; however, it can be made use of to identify how some of the clients' difficulties unfold in the “here and now” of the patient's daily life and relationships.

Typical goals of these therapies may include, i.e., reducing the patient's general symptoms: the therapist helps the client to reflect on identified difficulties in the patient's external reality (8). This may give way to the exploration of deeper dynamics and experiences, with the aim of improving the patient's resilience. Short-term psychoanalytical models [e.g., (9)] and short-lived psychodynamically-oriented treatments tend to focus first on the more urgent and important conflicts, unraveling the reasons as to why the patient sought a consultation. These conflicts are regarded as “focal” or “central” conflicts.

Brief psychodynamic therapies usually last between 20 and 40 sessions. Such treatments typically comprise three main stages: a beginning, a middle phase, and an end. Their treatment length can vary and can range between the higher and lower end of the continuum with regards to number of sessions offered. Brief psychodynamic therapies differ significantly from the classic psychoanalytical model, whereby an open-ended and more intensive psychoanalytic work is provided.

One feature that seems to further distinguish short-to-medium-term psychodynamic psychotherapies from more classic psychoanalytical treatments regards the use of transference, regarded as the process by which unconscious feelings and fantasies are transferred to the analyst (10). In brief psychodynamic treatments, this may be more diluted or made use of differently and less intensely than in more intensive therapies. That said, therapists may make use of their understanding of transference dynamics to work on the reason(s) for their patient's referral and the pattern(s) of their behavior and emotional responses (8, 11). Brief psychodynamic psychotherapy may not treat deeper anxieties or dynamics in the history of patients or their parents. The focus of short-term psychodynamic psychotherapy is rather confined to the “main anxiety,” and to the problem(s) that led the individual to seek therapeutic help, which may be a specific symptom or a specific relational dynamic.

The therapist holds important responsibilities about treatment planning, bearing in mind that its duration can be flexible, but can't be endless (8).

It is essential to plan the stages of treatment, if possible. In order to be able to do so, the therapist should preferably gain a good preliminary understanding of the patient's history right from their first meetings. Sometimes, an extended assessment is required in order to achieve a deeper level of understanding of the client's presentation.

Overall, there seem to be fewer published studies that focus on the effectiveness of psychodynamic psychotherapy with children and adolescents compared to the existing body of research focusing on the efficacy of cognitive-behavioral treatment approaches (12–14). However, more recently, an increasing demand for evidence-based treatments, outcome and process research, has triggered an interest in the way brief psychodynamic psychotherapies for children and young people operate (9).

The literature highlights how play therapy and psychodynamic therapy (15) can be effective for a broad array of psychological problems in children, including emotional and behavioral issues, post-traumatic disorders, and family and social problems (16). Recent studies have shown how beneficial psychodynamic therapy can be for young people, with improvements that typically persist after the end of the therapy (17, 18). However, the widely-perceived difficulties of engaging adolescents in psychotherapeutic work (i.e., high rates of adolescents' dropout, etc.) may have hindered the development of adolescent-focused models of time-limited therapy (19). Muratori et al. (20) examined the short- and long-term effects of time-limited psychodynamic psychotherapy for children with Internalizing disorders. They found the therapy useful on Internalizing symptoms in both the short and the long term, thanks to its sleeper effect (with a delayed onset). A review by Abbass et al. (21) generated encouraging results, and the authors concluded that psychodynamic psychotherapy is effective with adolescents. Based on studies that analyzed data recorded by means of well-validated symptom checklists, the authors found that, in all areas of interest, except for somatic symptoms, patients benefited significantly from the treatment by comparison with control groups, in both the short and medium term (21).

Family characteristics are significant predictors of a child's mental health; the emotional climate (family warmth), the family structure and its organization are regarded as having an impact or being associated with outcomes in children's psychotherapy treatments (22, 23).

Alongside an individual child psychodynamic and/or psychoanalytic psychotherapy, an area of good practice includes sessions of parallel parent work (24); furthermore the existing literature highlights how helpful and relevant it is to establish a good relationship with the family to promote the child's development, as highlighted by a number of authors (25–31).

Psychotherapy work with parents can influence the child's outcomes, when in treatment (32). High-quality, effective parenting support, and interventions have shown supportive of the psychotherapy process, by reducing the high prevalence of the emotional and behavioral problems among youth after treatment (33). It is estimated that children and young people present with higher risks of treatment drop-out as well as it is estimated that their family functioning is affected when the client's individual therapy is not associated to parallel parent work (34).

This study wishes to contribute to the existing body of literature evaluating the level of effectiveness of psychodynamic psychotherapies on symptoms' reduction of children and young people and on the level of family empowerment in two conditions (depending on whether the parents were offered parallel sessions or not).

### Aims

This outcome study, conducted in 2016 (from January to December), focused on evaluating the effectiveness of 1 year psychodynamic psychotherapy with children and adolescents. It was also aimed to assess whether there were different outcomes depending on whether parallel parents' sessions had been offered. It is important to emphasize that the present work is part of a broader, longitudinal study, conducted in the Child and Adolescent Neuropsychiatric Service, provided by the Italian National Health Service at a Local Mental Health Unit (ULSS 6) in Padua (PD, Italy). Aim of this present work was to use public resources to provide psychodiagnostic and therapeutic interventions in clinical practice, with the goal of identifying the most suitable psychotherapies for our service users. The study follows the official standards of clinical practice and research, as adopted by the scientific community, to improve the efficacy and effectiveness of psychotherapy for children and adolescents in mental health services. This research was conducted despite the challenges dictated by cuts to fundings for mental health Services in Italy.

The present project (approved by the local ethical committee—CEP 204 SC) was based on the above-mentioned premises, giving important consideration to the family household during the process of a patient's referral into the Service. The therapeutic approach involving the parents in the treatment considers the family as a structured subsystem and a composite set of different functions and roles, amongst which the roles of parenting, co-parenting, etc (35, 36).

Specifically, we examined: (a) the effectiveness of individual psychotherapy (with or without parallel work for their parents) on the children and adolescents' symptoms at the end of the therapy; (b) the relationship between the participants' individual psychotherapy (with or without parallel work for their parents) and the parents' perceptions of their parental empowerment.

We hoped to observe an improvement of the patient's symptomatology at the end of the therapy that would confirm the effectiveness of this mode of short-term psychodynamic psychotherapy with this population (37). Furthermore, we hypothesized that the child or adolescent's symptoms' reduction may also be associated with parents' perceptions of their parental empowerment.

## Materials and Methods

### Participants

The sample consisted of 57 families (each of them including one minor with two parents), who were referred to the Service during the course of 2016. The children and adolescents taking part in the study included 30 males and 27 females, aged between 6 and 18 years (*M* = 12.08, SD = 3.7). The wide age-range is justified by the nature of our clinical service, which accepts referrals to neuropsychiatric and psychotherapy services for both children and adolescents.

The sample was divided in two groups depending on the treatment offered, based on the participants' clinical condition and their carers'parenting skills: Group 1 (G1) included 26 participants (children and adolescents) who were offered individual therapy; and Group 2 (G2) consisted of 31 children and adolescents who received individual therapy, whose parents also received support alongside their child's psychotherapy.

Participants were assigned to one or the other group depending on the result of their assessment: if poor parenting/coparenting skills were found in addition to the client's psychopathology, the family was assigned to Group 2 (psychotherapy for the child/adolescent associated with parental support).

**Figure 1:**
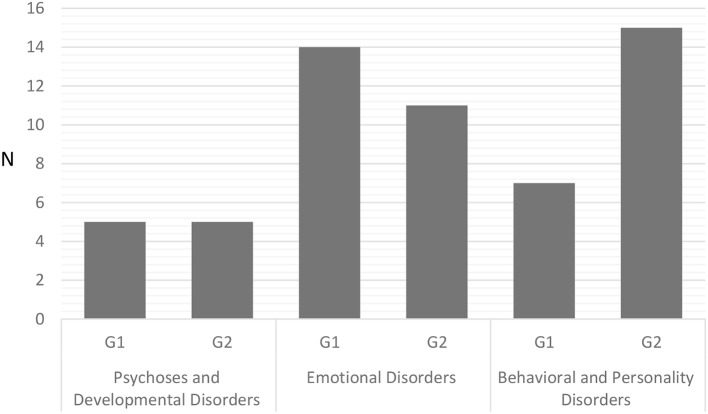
Number of participants in the three diagnostic categories and two groups. G1, Individual psychodynamic psychotherapy for child/adolescent; G2, Individual psychodynamic psychotherapy for child/adolescent and Co-parental support.

Each patient received a diagnosis on the ICD-10 (1). Depending on their ICD-10 diagnosis, participants were allocated to one of three macrocategories (see Figure 1): Psychoses and Developmental Disorders (1); Emotional Disorders (2); or Behavioral and Personality Disorders (3).

Category 1 (17%)—Psychoses and Developmental Disorders—involved: (F10–F19) Mental and behavioral disorders due to psychoactive substance use; (F80–F89) Disorders of psychological development.
Category 2 (44%)—Emotional Disorders—included: (F30–F39) Mood [affective] disorders; (F40–F48) Neurotic, stress-related and somatic disorders.
Category 3 (39%)—Mental and behavioral disorders—concerned: Personality Disorders (F60–F69) and Behavioral and emotional disorders with onset usually occurring in childhood and adolescence (F90–F98).

### Procedure

Our sample was recruited following an assessment with the children/adolescents and their parents. This assessment took place over a few meetings and interviews, depending on the need. Interviews were led and conducted by a developmental neuropsychiatrist and a trained psychodynamic psychologist. As part of the process, written consent for the child's therapy was sought at the time of referral. Parents also provided valid written consent for the use of video/audio recordings obtained during the sessions for research purposes.

The assessment procedure for the recruitment of our sample is outlined below.

- A first meeting was organized between the neuropsychiatrist and the child/adolescent, aimed to assess the patient's suitability for therapeutic intervention and/or psychiatric care. Following the above, two clinical interviews were conducted, and the child/young person was given a battery of tests; the Youth Self Report, YSR (38), was used at this stage of the assessment process. Then, a final feedback interview was conducted to inform the client and/or their parents of the ICD-10 diagnosis and discuss therapeutic recommendations.
- On a parallel level, the psychologist met separately with the parents. Subsequently, parents met the neuropsychiatrist, after which two clinical interviews were organized. The CBCL (Child Behavior Checklist) and the FES (Family Empowerment Scale) were administered at this stage of the process. Then, a final interview (which follows, in the next paragraph) provided feedback to the parents and their child.
- The final session, which involved the whole family, was organized and led by two professionals.

At this point of the assessment process, families were asked if they were willing to take part in this research project, following which a separate research consent form was signed. Our exclusion criteria for the present sample concerned a disability or an IQ <70, tested during the neuropsychological assessment using the WISC-III and/or WPPSI-III (39, 40).

### Study Design

This study was an outcome research. The sample was divided in two groups depending on treatment: one (Group 1) received 40 (weekly or fortnightly) sessions of individual Short-Term Psychodynamic Psychotherapy; the other (Group 2) received the same amount of individual Short-Term Psychodynamic Psychotherapy for the child with 20 (fortnightly or once/month) parallel parent sessions. The Short-Term Psychodynamic Psychotherapy in use is a time-limited psychodynamic psychotherapy that focuses on working through core problems and conflicts, also providing symptom relief [cfr. (41)]. This psychotherapeutic model is based on some key principles: (a) attention to the client-therapist relationship; (b) the therapist has an active role during treatment; (c) identification of a specific problem; (d) therapies have a time-limit and a fixed number of sessions.

Parent work was often helpful, considering the level of risk of the young person. It was conducted by a different therapist to the one working clinically with the child/young person; this is in keeping with studies confirming that families affected by multiple problems benefit greatly when parental support is offered alongside individual psychotherapy (42). The work with parents lasted for 12 months, and focused on three important areas, as suggested by Piovano (43): (a) the couple's relational triangulation; (b) the triangulation introduced by the child; and (c) the development of sufficiently good parenting functions. Therapists met periodically for supervision, to discuss cases and share therapeutic objectives.

**Figure 2:**
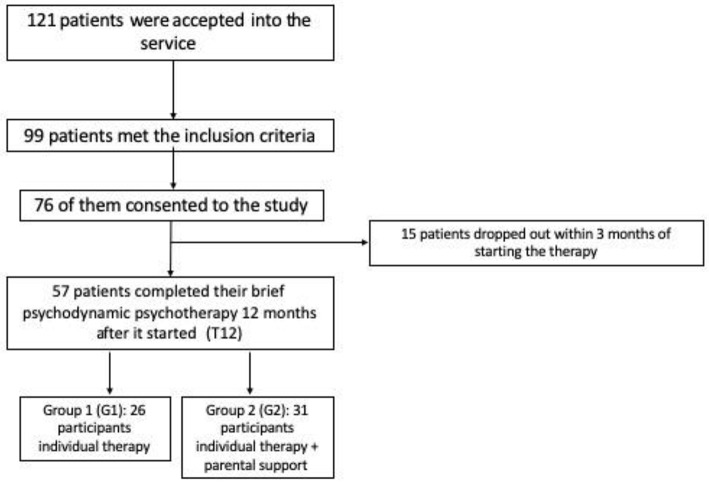
Flow-diagram of study design.

Out of 141 referrals, 121 patients were accepted into the service; 99 of them met our inclusion criteria, and 76 consented to the study. Fifty-seven patients completed their brief psychodynamic psychotherapy 12 months after it started, while 19 did not attend (15 dropped out within 3 months of starting the therapy because they reported they no longer needed treatment, 2 chose another service, and 2 moved out of area) (see Figure 2).

### Instruments

#### Child Behavior Checklist (CBCL) and Youth Self Report (YSR) [(38); It. Tr. (44)]

These well-validated questionnaires are adopted worldwide and are commonly used to assess behavioral and emotional difficulties in children and young people. Children's parents completed the CBCL, and—for the present study—both parents were asked to answer the questionnaire jointly, considering the last 6 months of their child's life. The YSR was administered to adolescents between 11 and 18 years of age.

Raw answers to the questionnaires were scored using the computer-based Assessment Data Manager (ADM) program, part of the Achenbach System for Empirically-Based Assessments (ASEBA) (38), which produces a clinical profile in the form of a set of scales referring to specific symptom domains. These domains identify the following syndromes: anxiety/depression, withdrawn behavior, somatic complaints, social problems, thought problems, attention problems, aggressive behavior, and rule-breaking behavior. A further area of the profile illustrates three clusters of issues, identifiable as: Internalizing, Externalizing and Total Problems. Internalizing problems include anxiety/depression, withdrawn behavior and somatic complaints. Externalizing problems involve aggressive behavior and rule-breaking behavior. Total Problems are a combination of both Internalizing and Externalizing Problems, and any Other problems, such as tics, suicidal ideation, pica, weight-related problems, speech problems, etc.

For the present study, only the three main clusters were considered, i.e., Internalizing, Externalizing and Total Problems. Scores obtained on these scales were rated in terms of their clinical severity as non-clinical, borderline, or clinical, using cut-offs: scores of 64 or more were regarded as “clinical,” scores between 60 and 63 as “borderline,” and scores of 59 or less as “non-clinical.” Several studies confirmed the reliability and validity of the Italian versions of both CBCL and YSR (45, 46). In particular Frigerio et al. (44) observed very-good Cronbach α coefficients in CBCL scales ranging from 0.83 to 0.91.

#### Family Empowerment Scale [FES- (47)]

This is a brief questionnaire designed to assess family members' perceptions of empowerment. The 34 items on the FES tap into two dimensions of family empowerment: level of empowerment (family, service system, community/political); and how empowerment is expressed (attitudes, knowledge, behavior). Given the focus of our study, only the family subscale (12 items) referring to parents' management of everyday situations was used. Answers are given on a Likert scale and range from “never” (1) to “very often” (5). Total scores range from 12 to 60, and there is no cut-off. The Italian version shows very good reliability reporting a McDonald's ω of 0.846 and 0.832 for Mothers' and Fathers' sub-scales, respectively.

The use of this indicator of internal consistency is in line with recent literature about the critical aspects related to the use of Cronbach's α [e.g., (48)]. McDonald's ω appears to be a more appropriate index of the extent to which the items of a test measure the same latent variable [e.g., (49–51)]. The values of this coefficient are interpreted similarly to those of Cronbach's α, but they are not affected by the same weaknesses.

Questionnaires CBCL and YSR were part of the current clinical practice; they were administered before and after the psychotherapy. The FES has been identified for research intent.

### Statistical Analysis

All the analyses were conducted using “JASP 0.9” software (52), along with descriptive statistics aiming to provide a clearer picture of the sample. In order to test our research questions, several mixed model ANOVAs were run with repeated measures: to test *time* as a “within factor” (2 levels: T0 and T12 after 1 year of treatment) and to test the *type of treatment* as a “between factor” (2 levels: individual psychotherapy for the child vs. individual psychotherapy for the child combined with parallel parent sessions).

## Results

### Child/Adolescent Psychopathology

**Figure 3:**
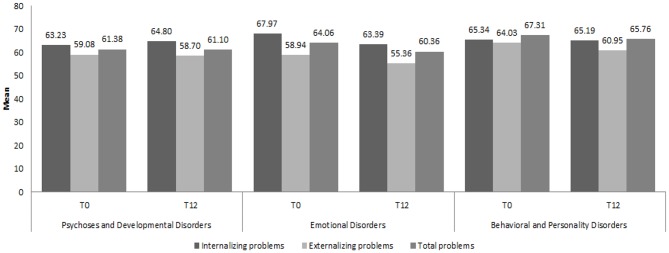
Mean scores of the three Child Behavior Checklist scales by diagnostic category at T0 and T12. CBCL, Child Behavior Checklist (38); T0, Time of First evaluation, during clinical assessment; T12, Time of *Final* evaluation, after 12 months of brief psychodynamic treatment.

**Figure 4:**
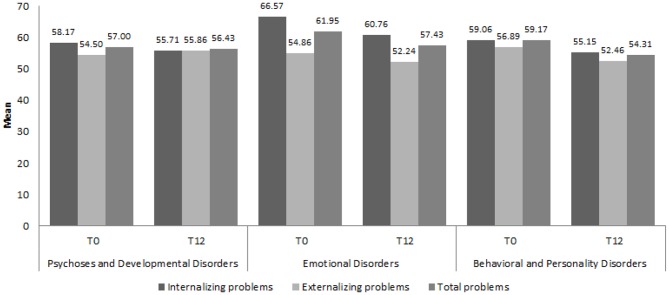
Mean scores of the three Youth Self Report scales by diagnostic category at T0 and T12. YSR, Youth Self Report (38); T0, Time of First evaluation, during clinical assessment; T12, Time of *Final* evaluation, after 12 months of brief psychodynamic treatment.

Figures 3, 4 show the distribution of the mean CBCL and YSR scores, respectively, for Internalizing, Externalizing and Total Problems at T0 and T12, by diagnostic category.

**Figure 5:**
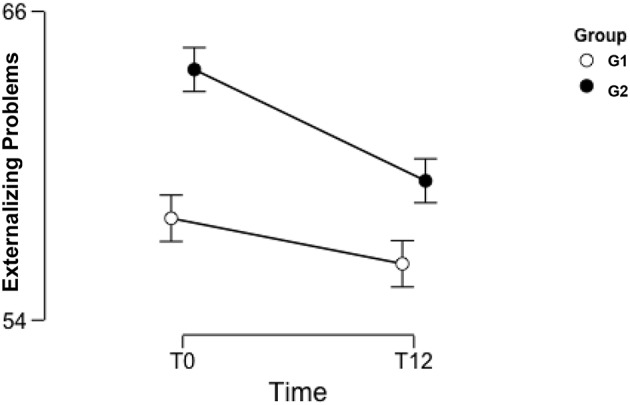
Mean of Child Behavior Checklist scores for Externalizing Problems in G1 and G2 at T0 and T12. CBCL, Child Behavior Checklist (38); T0, Time of First evaluation, during clinical assessment; T12, Time of *Final* evaluation, after 12 months of brief psychodynamic treatment. G1, Individual psychodynamic psychotherapy for child/adolescent; G2, Individual psychodynamic psychotherapy for child/adolescent and Co-parental support.

The mixed model ANOVA on the main scores on the CBCL highlighted a significant effect of both therapies in reducing the severity of the problems in all the investigated areas. The main effect of the within factor “Time” was significant for the three subscales Internalizing Problems [F_(1, 55)_ = 12.142; *p* ≤ 0.001, η^2^ = 0.177], Externalizing Problems [*F*_(1, 55)_ = 11.959; *p* ≤ 0.001, η^2^ = 0.173], and Total Problems [*F*_(1, 55)_ = 20.144; *p* ≤ 0.001, η^2^ = 0.265]. None of the interactions between the two factors were significant, indicating a substantially equivalent effect of the different therapies over time. The main effect of the factor “Group” was significant only with respect to the Externalizing Problems scale [*F*_(1, 55)_ = 4.018; *p* = 0.05, η^2^ = 0.068]. This last result suggests that participants in G1 significantly differed from participants in G2 concerning the level of their Externalizing problems throughout the observation period (see Figure 5).

Table 1 (below) shows the two groups' mean scores for Internalizing, Externalizing and Total Problems at T0 and T12. It is worth noticing that, for Externalizing Problems, the mean score for G1 at T0 is in the non-clinical range, while, for G2, this is in the clinical range. All the other pairs of measures were both within the same range for severity.

**Table 1:** Mean scores of the three Child Behavior Checklist scales for each group at T0 and T12.

		**Internalizing problems**		**Externalizing problems**		**Total problems**		**Participants**
**Time**	**Group**	***Mean***	***SD***	***Mean***	***SD***	***Mean***	***SD***	***N***
T0	G1	68.38	6.940	57.96	9.327	65.31	8.054	26
	G2	67.16	7.975	63.74	8.862	66.71	7.230	31
T12	G1	66.23	9.132	56.19	8.859	62.38	9.113	26
	G2	62.58	9.284	59.42	9.248	62.35	9.496	31

*CBCL, Child Behavior Checklist (38); T0, Time of First evaluation, during clinical assessment; T12, Time of Final evaluation, after 12 months of brief psychodynamic treatment. G1, Individual psychodynamic psychotherapy for child/adolescent; G2, Individual psychodynamic psychotherapy for child/adolescent and Co-parental support*.

A smaller group of adolescent patients (between 11 and 18 years old) completed the YSR 11–18 at T0 and T12. Table 2 shows a descriptive analysis of the three YSR scales for Internalizing, Externalizing and Total Problems for Groups 1 and 2 at the two time points.

**Table 2:** Mean scores of the three Youth Self Report scales for each group at T0 and T12.

		**Internalizing problems**		**Externalizing problems**		**Total problems**		**Participants**
**Time**	**Group**	***Mean***	***SD***	***Mean***	***SD***	***Mean***	***SD***	***N***
T0	G1	64.56	10.94	52.25	10.529	59.88	11.581	16
	G2	61.84	10.77	56.47	9.518	60.32	9.855	19
T12	G1	58.94	12.43	52.31	11.359	57.06	12.124	16
	G2	58.58	10.55	53.84	9.895	56.58	9.963	19

*YSR, Youth Self Report (17); T0, Time of First evaluation, during clinical assessment; T12, Time of Final evaluation, after 12 months of brief psychodynamic treatment. G1, Individual psychodynamic psychotherapy for child/adolescent; G2, Individual psychodynamic psychotherapy for child/adolescent and Co-parental support*.

The results of the ANOVA showed a significant change in YSR scores between T0 and T12 with regards to the Internalizing Problems [*F*_(1, 55)_ = 11.580; *p* = 0.002, η^2^ = 0.255] and the Total Problems [*F*_(1, 55)_ = 7.551; *p* = 0.010, η^2^ = 0.186] scales. No effect of the between factor emerged, indicating that there were no significant differences between the two treatment groups. None of the interactions between the two factors reached significance, indicating a substantially homogeneous trend over time in the reduction of the problems in both groups.

### Family Empowerment

Table 3 shows mothers and fathers' scores on the FES, for both groups. The results revealed no statistically significant change in the sample's perception of sense of empowerment between T0 and T12.

**Table 3:** Mean scores and Standard deviation of the Family Empowerment Scale for both parents of participants in G1 and G2.

		**FES mothers**		**FES fathers**		**Participants**
**Time**	**Group**	***Mean***	***SD***	***Mean***	***SD***	**N**
T0	G1	44.25	5.290	44.33	4.419	20
	G2	43.67	6.983	42.20	4.950	27
T12	G1	44.40	4.672	43.33	5.367	20
	G2	44.63	5.603	43.16	5.632	27

*FES, Family Empowerment Scale (47); T0, Time of First evaluation, during clinical assessment; T12, Time of Final evaluation, after 12 months of brief psychodynamic treatment. G1, Individual psychodynamic psychotherapy for child/adolescent; G2, Individual psychodynamic psychotherapy for child/adolescent and Co-parental support*.

## Discussion

This outcome study yielded some preliminary and non-generalizable findings on the effects of this time-limited psychodynamic psychotherapy with a population of young people aged 6–18, sampled in a local Mental Health Unit in Northern Italy. This study gave us the opportunity to examine the area of presenting symptoms before and after therapy, in two groups, when parents received vs. did not receive therapeutic support on a fortnightly basis. Measures of the effects of treatment were the levels of reported symptoms by the patients and their parents and the level of parents' perception of the family empowerment.

Statistical analyses showed significant reductions in the CBCL scores in the areas of Internalizing, Externalizing and Total Problems at T12, compared to T0; the YSR scores also showed improvements in the areas of Internalizing and Total Problems as reported by the patients.

Despite the initially encouraging results of this 1 year-long outcome study, one may have to thread carefully with their interpretation.

Prior to starting therapy, at baseline, Group 2 revealed a more severe clinical profile than Group 1 in the area of Externalizing Problems (i.e., aggressive behavior, oppositional and conduct disorders etc.), as shown by the results obtained on the Achenbach's questionnaires, CBCL and YSR 11–18. The offer of parent work was motivated by their clinical presentation at the moment of referral, with some families presenting with difficulties in their parenting. Although we are not bound to know exactly what the relationship between parenting difficulties and the presence of Externalizing symptoms in children and youth may be, it may be possible that poor parenting (i.e., characterized by lack of warmth, rigid and/or negative parenting style, poor monitoring of the children, etc.) is directly associated with Externalizing problems in childhood.

After 12 months of treatment, the scores obtained by Group 2 in Externalizing symptoms (on both the CBCL and the YSR) showed a statistically significant clinical improvement, which is encouraging; however, because of the presence of a statistically significant difference at baseline between the two groups in the area of Externalizing Problems, results are not immediately comparable in this area because the two groups did not present with similar levels of Externalizing difficulties at the onset and throughout the treatment.

Our findings also seem to highlight that, with this population, brief psychodynamic therapy seemed to be effective on symptoms' reduction with regards to Internalizing symptoms, as reported by the clients and their parents. It is appreciated in literature that psychodynamically-oriented therapies seem to be most effective with children and young people affected by Internalizing difficulties. It is possible that children and young people presenting with internalizing difficulties improve their insight about their difficulties thanks to being in treatment (18). Further studies could investigate which internal or psychotherapeutic processes occur and facilitate this growing capacity in children and adolescents, in order to evaluate what works best and for whom (53).

The psychodynamic approach to therapy enhances exploration and reflection on the client's emotional sphere, their affects and thoughts. The literature seems to highlight that time-limited psychodynamic psychotherapies are less effective on Externalizing symptoms and it may be possible that different, multimodal approaches (54–57) or mixed treatment approaches—including cognitive-behavioral techniques—are needed with this array of difficulties from the onset of treatment (58, 59).

Further, working clinically with parents requires a high level of experience and presents with major challenges: parents often require both emotional containment and practical advice on how to manage their child's behaviors and may need more time to improve their relationship with their children/adolescents depending on their internal and interpersonal resources. An added layer of complexity while working clinically with young people and their parents in this study was represented by the variety of ICD-10 diagnostic categories of this sample. Our findings seemed to point toward positive changes and outcomes in the CBCL and YSR scores of children and young people affected by “Emotional Disorders” and “Behavioral and Personality Disorders,” whilst no positive changes were evaluated on questionnaires in the “Psychoses and Developmental Disorders” category.

Our results seem to partly support the hypothesis according to which psychodynamic psychotherapy might not be as effective as other approaches in treating such disorders, whereas it constitutes an eligible treatment for depression, anxiety, eating disorders, somatic, and personality disorders (60). With these regards, a study by Gonzalez (61) evaluated that psychodynamic psychotherapy seemed to be effective only on the depressive symptoms of clients affected by bipolar disorder.

Furthermore, our results showed a discrepancy between the rates of Internalizing problems (expressed in percentages) as reported by parents and as reported by their children: the YSR scores suggested higher rates of Internalizing Problems at Time 0 (35%) compared to the CBCL scores (24%). On the whole, parents are reported in literature to be better equipped to recognize Externalizing problems in their children's behavior because these are more visible than internal problems or intrapsychic difficulties. Internalizing problems might also be more socially acceptable because of the limited impact they have on the outside world (62).

The parents' supportive intervention offered in the Neuropsychiatric Service aimed to help parents recognize their child's and their own emotional difficulties (63, 64). Whilst the children and adolescents' psychopathology had improved after therapy; in our study, adults' parenting skills did not seem to follow the same trend and no positive change was evaluated in the domain of *family empowerment*. This result may be motivated by a number of reasons. It may be that increasing parents' awareness of specific issues might prompt a sense of incompetence and guilt, independently from their children's clinical outcome. Interestingly, parents reported greater improvements, in their children's symptoms, than their children 12 months after treatment. Furthermore, it would have been helpful to explore if feelings linked to ending the treatment impacted parents' ratings on the *Family Empowerment Scale*.

It may also be important to consider that clients presented with high levels of comorbidity at referral and received an ICD-10 diagnosis following their assessment. It was not possible to evaluate the impact of receiving a diagnosis on the family nor on their children's symptoms' improvements and it is hoped that further qualitative work will explore the impact that this may have on the family's perception of empowerment.

Based on findings of existing literature, parents of children with behavioral or emotional difficulties seem to experience lower levels of self-efficacy than parents of children/adolescents who are not affected by mental health issues (65). Parent psychodynamic work can be highly beneficial in supporting child/adolescent psychotherapy, after an initial period of adjustments and adaptation has been made.

It is relevant to consider that a self-report measure might not capture the nuances of what is defined as clinical change and improvement in parent work, not accounting for the family's history, nor for their current relationship dynamics or difficulties. Given the risk of adopting a reductivist approach to the dimension of change in psychodynamic psychotherapy with children, young people and their families, more in depth/qualitative research would be helpful in studying what leads to change both in individual and in parents' therapeutic work and how to capture it. As Whitefield and Midgley suggest, “working with parents' histories in parent work, however, where parents are attending sessions without their child, and yet not as patients themselves, may bring with it particular challenges” [(24), p. 273]. Systemic and psychoanalytic theories seem to agree in saying that homeostatic influences and resistances can occur when working clinically with families; change in one or the other parent could affect the couple's relationship, as well as their sense of empowerment (66).

## Conclusion

It is important to highlight that this outcome study has attempted to capture information on the symptoms of a clinical population seen in a local Mental Health Service in Northern Italy to evaluate whether these symptoms had improved after 12 months of short-term-psychodynamic psychotherapy. The use of well-validated self-report measures was essential but felt limited to T0 and T12. It would have been useful to collect data *in itinere*, and thus draw comparisons that would shed light on *how* (and not only *if*) our young participants responded to the treatment. The authors recognize that self-report questionnaires are susceptible to psychological biases and can be under the influence of social desirability.

Further, the way participants were assigned to each condition of the study—namely based on their clinical presentations and profiles—prevented any randomization and no causal relationships between the variables could be inferred from this research.

Despite these limitations, our results seem to demonstrate an overall effectiveness, on symptoms' reduction, of our time-limited psychodynamic psychotherapy in treating children and adolescents with psychopathological issues. This study is rooted in the real-world experience of clinical practice and therefore may present with important strengths. Its preliminary findings contribute to the growing body of literature on the use and the effectiveness of short-term psychodynamic psychotherapy with children and adolescents for a variety of psychiatric diagnoses (20, 67–69). Mindful that an outcome study is the starting line for future research on the topic, this study's findings add to the growing evidence calling for more tailored and bespoke interventions for children and adolescents. This is based on the view that a child's development is the product of a varied and dynamic interaction between closely-interwoven factors, including co-parenting and the child's treatment within the family (70).

## Data Availability Statement

The raw data supporting the conclusions of this article will be made available by the authors, without undue reservation, to any qualified researcher.

## Ethics Statement

Ethical-Committee approval CEP 204 SC.

## Consent

The authors declare that written informed consent was obtained from all patients (or other parties) before their participation in the study, which had obtained the prior approval of the Ethical Committee of the ULSS 16.

## Author Contributions

MG, MM, LS, AS, MD, MS, and EF have given a substantial contribution to the conception and implementation of the work, taking part to data acquisition, analysis and discussion, drafting, and revising the manuscript. All authors revised and reached an agreement on the final version of the work.

### Conflict of Interest

The authors declare that the research was conducted in the absence of any commercial or financial relationships that could be construed as a potential conflict of interest.

## References

[1] World Health Organization ICD-10: International Statistical Classification of Diseases and Related Health Problems: Tenth Revision. 2nd ed. World Health Organization (2004). Available online at: https://apps.who.int/iris/handle/10665/42980

[2] Gabbard GO Long-Term Psychodynamic Psy-chotherapy: A Basic Text. 3rd ed. Arlington, VA: American Psychiatric Publishing (2017).

[3] Briggs S Maxwell M Keenan A Working with the complexities of adolescent mental health problems: applying time-limited adolescent psychodynamic psychotherapy (TAPP). Psychoanal Psychother. (2015) 29:314–29. 10.1080/02668734.2015.1086414

[4] Rossouw TI Fonagy P. Mentalization-based treatment for self-harm in adolescents: a randomized controlled trial. J Am Acad Child Adolesc Psychiatry. (2012) 51:1304–13. 10.1016/j.jaac.2012.09.018 23200287

[5] Lemma A Target M Fonagy P Brief Dynamic Interpersonal Therapy: A Clinician's Guide. New York, NY: Oxford University Press (2011).

[6] Midgley N Cregeen S Hughes C Rustin M. Psychodynamic psychotherapy as treatment for depression in adolescence. Child Adolesc Psychiatr Clin N Am. (2013) 22:67–82. 10.1016/j.chc.2012.08.004 23164128

[7] Trowell J Joffe I Campbell J Clemente C Almqvist F Soininen M. Childhood depression: a place for psychotherapy. Eur Child Adolesc Psychiatry. (2007) 16:157–67. 10.1007/s00787-006-0584-x 17200793

[8] Rawson P Short-Term Psychodynamic Psychotherapy: An Analysis of the Key Principles. London: Karnac Books (2002).

[9] Goodyer IM Tsancheva S Byford S Dubicka B Hill J Kelvin R Improving mood with psychoanalytic and cognitive therapies (IMPACT): a pragmatic Effectiveness superiority trial to investigate whether specialized psychological treatment reduces the risk for relapse in adolescents with moderate to severe unipolar depression: Study protocol for a randomized controlled trial. Trials. (2011) 12:175 10.1186/1745-6215-12-175 21752257 PMC3148993

[10] Zepf S The psychoanalytic process and Freud's concepts of transference and transference neurosis. Psychoanal Psychol. (2010) 27:55–73. 10.1037/a0018640

[11] Cregeen S Short-Term Psychoanalytic Psychotherapy for Adolescents With Depression: A Treatment Manual. London: Routledge (2018).

[12] Fonagy P Target M Regolazione Affettiva, Mentalizzazione e Sviluppo del sé. Milano: Raffaello Cortina Editore (2005).

[13] Hibbs ED. Evaluating empirically based psychotherapy research for children and adolescents. Eur Child Adolesc Psychiatry. (2001) 10:1/3–1/1. 10.1007/s007870170002 11794555

[14] Weisz JR Kazdin AE Evidence-Based Psychotherapies for Children and Adolescents. New York, NY: Guilford Press (2010).

[15] Edlund JN Carlberg G Psychodynamic psychotherapy with adolescents and young adults: outcome in routine practice. Clin Child Psychol Psychiatry. (2016) 21:66–80. 10.1177/1359104514554311 25326532

[16] Leblanc M Ritchie M A meta-analysis of play therapy outcomes. CounsPsychol Q. (2001) 14:149–63. 10.1080/09515070110059142

[17] Abbass AA Rabung S Leichsenring F Refseth JS Midgley N. Psychodynamic psychotherapy for children and adolescents: a meta-analysis of short-term psychodynamic models. J Am Acad Child Adolesc Psychiatry. (2013) 52:863–75. 10.1016/j.jaac.2013.05.014 23880496

[18] Midgley N Kennedy E Psychodynamic psychotherapy for children and adolescents: a critical review of the evidence base. J Child Psychother. (2011) 37: 232–60. 10.1080/0075417X.2011.614738

[19] Briggs S Lyon L A developmentally focused time-limited psychodynamic psychotherapy for adolescents and young adults: origins and application. Adolescence. (2011) 76:415–34. 10.3917/ado.076.0415

[20] Muratori F Picchi L Bruni G Patarnello M Romagnoli G. A two-year follow-up of psychodynamic psychotherapy for Internalizing disorders in children. J Am Acad Child Adolesc Psychiatry. (2003) 42:331–9. 10.1097/00004583-200303000-00014 12595787

[21] Abbass AA Kisely SR Town JM Leichsenring F Driessen E De Maat S Short-term psychodynamic psychotherapies for common mental disorders. Cochrane Database Syst Rev. (2014) CD004687. 10.1002/14651858.CD004687.pub4 PMC11129844 24984083

[22] McBride BA Schoppe-Sullivan SJ Ho MH The mediating role of fathers'school involvement on student achievement. J. Appl Dev Psychol. (2005) 26:201–16. 10.1016/j.appdev.2004.12.007

[23] Bergin C Bergin D Attachment in the classroom. Educ. Psychol Rev. (2009) 21:141–70. 10.1007/s10648-009-9104-0

[24] Whitefield C Midgley N ‘And when you were a child?': how therapists working with parents alongside individual child psychotherapy bring the past into their work. J Child Psychother. (2015) 41:272–92. 10.1080/0075417X.2015.1092678

[25] Gatta M Balottin L Mannarini S Chesani G Del Col L Spoto A Familial factors relating to alexithymic traits in adolescents with psychiatric disorders. Clin Psychol. (2016) 21:252–62. 10.1111/cp.12098

[26] Gatta M Miscioscia M Sudati L Sisti M Comis I Battistella BA. Contribution of analyses on triadic relationships to diagnostics and treatment planning in developmental psychopathology. Psychol Rep. (2017) 120:290–304. 10.1177/0033294116688454 28558624

[27] Mannarini S Balottin L Munari C Gatta M Assessing conflict management in the couple: the definition of a latent dimension. Family J. (2016) 25:13–22. 10.1177/1066480716666066

[28] Gatta M Miscioscia M Sisti M Comis I Battistella PA. Interactive family dynamics and non-suicidal self-injury in psychiatric adolescent patients: a single case study. Front Psychol. (2017) 8:1–5. 10.3389/fpsyg.2017.00046 28220084 PMC5292625

[29] Feinstein NR Fielding K Uduari-Solner A Joshi SV. The supporting alliance in child and adolescent treatment: enhancing collaboration among therapists, parents and teachers. Am J Psychother. (2009) 63:319–44. 10.1176/appi.psychotherapy.2009.63.4.319 20131741

[30] Novick KK Novick J. A new model of techniques for concurrent psychodynamic work with parents of child and adolescent psychotherapy patients. Child Adolesc Psychiatr Clin N Am. (2013) 22:331. 10.1016/j.chc.2012.12.005 23538016

[31] Robbins MS Turner CW Alexander JF Perez GA. Alliance and dropout in family therapy for adolescents with behavior problems: individual and systemic effects. J Fam Psychol. (2003) 17:534–44. 10.1037/0893-3200.17.4.534 14640803

[32] Karckay AT The relationship between parental attitude and social comparison of the eight grade students. Procedia Soc Behav Sci. (2009) 1:1469–73. 10.1016/j.sbspro.2009.01.259

[33] Shapiro CJ. Behavioral kernels and brief interventions: teaching parents effective behavior management strategies. N C Med J. (2013) 74:57–9. 23530384

[34] Midgley N O'Keeffe S French L Kennedy E Psychodynamic psychotherapy for children and adolescents: an updated narrative review of the evidence base. J. Child Psychother. (2017) 40:1–23. 10.1080/0075417X.2017.1323945

[35] Miscioscia M Simonelli A Svanellini L Sisti M Sudati L Brianda M An integrated approach to child psychotherapy with co-parental support: a longitudinal outcome study. Res Psychother: Psychopathol Proc Outcome. (2018) 21. 10.4081/ripppo.2018.297 PMC7451302 32913760

[36] Gatta M Sisti M Sudati L Miscioscia M Simonelli A The Lausanne Trilogue Play within the outcome evaluation in infant mental health: a preliminary report. Res. Psychother. Psychopathol. Process Outcome. (2016) 19. 10.4081/ripppo.2016.198

[37] Lis A Zennaro A Mazzeschi C Child and adolescent empirical psychotherapy research: a review focused on cognitive-behavioral and psychodynamic-informed psychotherapy. Eur Psychol. (2001) 6:36–64. 10.1027//1016-9040.6.1.36

[38] Achenbach TM Rescorla LA Manual for ASEBA School-Age Forms & Profiles. Burlington: University of Vermont, Research Center for Children, Youth and Families (2001).

[39] Wechsler D WISC-III: Wechsler Intelligence Scale for Children. New York, NY: The Psychological Corporation (1991).

[40] Wechsler D Wechsler Preschool and Primary Scale of Intelligence – Revised. San Antonio, TX: The Psychological Corporation (1989).

[41] Gabbard GO Del Corno F Lingiardi V (Eds.). Le Psicoterapie: Teorie e Modelli D'Intervento. Milano: Raffaello Cortina (2010).

[42] Chinitz S Guzman H Amstutz E Kohchi J Alkon M. Improving outcomes for babies and toddlers in child welfare: a model for infant mental health intervention and collaboration. Child Abuse Neglect. (2017) 70:190–8. 10.1016/j.chiabu.2017.05.015 28622589

[43] Piovano B Parenthood and parental functions as a result of the experience of parallel psychotherapy with children and parents. Int. Forum Psychoanal. (2004) 13:187–200. 10.1080/08037060410000650

[44] Frigerio A Cattaneo C Cataldo MG Schiatti A Molteni M Battaglia M Behavior and emotional problems among Italian children and adolescents aged 4 to 18 years as reported by parents and teachers. Eur J Psychol Assess. (2004) 20:124–33. 10.1027/1015-5759.20.2.124

[45] Ivanova M Achenbach T Dumenci L Rescorla L Almqvist F Weintraub S. Testing the 8-syndrome structure of the Child Behavior Checklist in 30 societies. J Clin Child Adolesc Psychol. (2007) 36:405–15. 10.1080/15374410701444363 17658984

[46] Ivanova MY Achenbach TM Rescorla LA Dumenci L Almqvist F Bilenberg N. The generalizability of the youth self-report syndrome structure in 23 societies. J Consult Clin Psych. (2007) 75:729–38. 10.1037/0022-006X.75.5.729 17907855

[47] Koren PE De Chillo N Friesen BJ Measuring empowerment in families whose children have emotional disabilities: a brief questionnaire. Rehabil Psychol. (1992) 37:305–21. 10.1037/h0079106

[48] Peters G-JY The alpha and the omega of scale reliability and validity: why and how to abandon Cronbach's alpha and the route towards more comprehensive assessment of scale quality. Eur Health Psychol. (2014) 16:56–69. 10.31234/osf.io/h47fv

[49] McDonald RP Test Theory: A Unified Treatment. Mahwah, NJ: Psychology Press (1999).

[50] Zinbarg R Revelle W Yovel I Li W Cronbach's α, Revelle's β, and McDonald's ωH: their relations with each other and two alternative conceptualizations of reliability. Psychometrika. (2005 70:123–33. 10.1007/s11336-003-0974-7

[51] Zinbarg R Yovel I Revelle W McDonald R Estimating generalizability to a universe of indicators that all have an attribute in common: a comparison of estimators for. Appl Psychol Meas. (2006) 30:121–44. 10.1177/0146621605278814

[52] JASP Team JASP (Version 0.9) [Computer Software] (2018).

[53] Roth A Fonagy P What Works For Whom?: A Critical Review of Psychotherapy Research. New York, NY: Guilford Press (2006).

[54] Stone LL Otten R Engels RC Kuijpers RC Janssens JM Relations between internalizing and externalizing problems in early childhood. In: Child & Youth Care Forum. Vol. 44. Springer US. (2015). p. 635–53.

[55] Johnston C Chronis-Tuscano A Families and ADHD. In: Barkley RA, editor. Attention-de cit/Hyperactivity Disorder: A Handbook for Diagnosis and Treatment. New York, NY: The Guilford Press (2014). p. 191–209.

[56] Stormshak EA Bierman KL McMahon RJ Lengua LJ. Parenting practices and child disruptive behavior problems in elementary school. J Clin Child Psychol. (2000) 29:17–29. 10.1207/S15374424jccp2901_3 10693029 PMC2764296

[57] Loginova SV Slobodskaya HR The mediating role of parenting in the relation between personality and externalizing problems in Russian children. Pers Individ Dif. (2016) 106:275–80. 10.1016/j.paid.2016.10.055

[58] Luborsky L McLellan AT Woody GE O'Brien CP Auerbach A. Therapist success and its determinants. Arch Gen Psychiatry. (1985) 42:602–11. 10.1001/archpsyc.1985.01790290084010 4004503

[59] Papoušek M Wollwerth de Chuquisengo R Integrative kommunikationszentrierteEltern-kleinkind-psychotherapiebeifrühkindlichenregulationsstörungen. Prax Kinderpsychol Kinderpsychiatr. (2006) 55:235–54. 17436558

[60] Fonagy P. The effectiveness of psychodynamic psychotherapies: An update. World Psychiatry. (2015) 14:137–50. 10.1002/wps.20235 26043322 PMC4471961

[61] Gonzales JM A case study of psychodynamic psychotherapy for bipolar disorder. Am J Psychother. (2007) 61:405–22. 10.1176/appi.psychotherapy.2007.61.4.405 18251385

[62] Kazdin AE Parent Management Training: Treatment for Oppositional, Aggressive, and Antisocial Behavior in Children and Adolescents. New York, NY: Oxford University Press (2005).

[63] Gatta M Dal Zotto L Nequinio G Del Col L Sorgato R Ceranto G Parents of adolescents with mental disorders: Improving their caregiving experience. J Child Fam Stud. (2011) 20:478–90. 10.1007/s10826-010-9415-2

[64] Gatta M Ramaglioni E Lai J Svanellini L Toldo I Del Col L. Psychological and behavioral disease during developmental age: the importance of the alliance with parents. Neuropsychiatr Dis Treat. (2009) 5:541–6. 10.2147/NDT.S5880 19898668 PMC2773285

[65] Sanders MR Woolley ML. The relationship between maternal self-efficacy and parenting practices: implications for parent training. Child Care Health Dev. (2005 31:65–73. 10.1111/j.1365-2214.2005.00487.x 15658967

[66] Sutton A Hughes L The psychotherapy of parenthood: towards a formulation and valuation of concurrent work with parents. JChild Psychother. (2005) 31:169–88. 10.1080/00754170500221253

[67] Goldfried MR Wolfe BE. Toward a more clinically valid approach to therapy research. J Consult Clin Psychol. (1998) 66:143. 10.1037/0022-006X.66.1.143 9489268

[68] Leichsenring F Rabung S. Effectiveness of long-term psychodynamic psychotherapy: a meta-analysis. JAMA. (2008) 300:1551–65. 10.1001/jama.300.13.1551 18827212

[69] Gatta M Canetta E Zordan M Spoto A Ferruzza E Manco I. Alexithymia in juvenile primary headache sufferers: a pilot study. J Headache Pain. (2011) 12:71–80. 10.1007/s10194-010-0248-6 20730593 PMC3072508

[70] Lindahl KM Methodological issues in family observational research. In: Kerig PK Lindahl KM, editors. Family Observational Coding Systems: Resources for Systemic Research. Philadelphia, PA: Brunner/Mazel (2001). p. 23–32.

